# Letter to the Editor: comment on “Thoracoscopic surgical ablation for atrial fibrillation patients with functional regurgitation the treatment strategy prioritizing atrial fibrillation”

**DOI:** 10.1097/JS9.0000000000003124

**Published:** 2025-08-07

**Authors:** Tan Liu, Yucheng Hou, Zhenya Shen

**Affiliations:** Department of Cardiovascular Surgery, The First Affiliated Hospital of Soochow University, Suzhou, China

*Dear Editor,*

We read with great interest the recent article by Sun *et al* titled “Thoracoscopic surgical ablation for atrial fibrillation patients with functional regurgitation: the treatment strategy prioritizing atrial fibrillation,” published in the *International Journal of Surgery*^[1]^. The authors reported that thoracoscopic surgical ablation (TSA) effectively eliminates atrial fibrillation (AF) and reduces functional regurgitation (FR). However, we believe several key aspects of this study merit further discussion. This correspondence adheres to the TITAN Guidelines 2025 – governing declaration and use of AI^[2]^.

First, FR is a recognized risk factor for AF and significantly increases the probability of AF recurrence. Conversely, AF can cause enlargement of both the left and right atria, leading to dilation of the mitral and tricuspid annuli, which may result in or aggravate FR^[3,4]^. This study demonstrates that TSA restores sinus rhythm (SR) and decreases FR severity – findings consistent with previous observations reported by Dhont *et al*^[4]^. Notably, at the 12-month follow-up after TSA, 102 out of 152 patients (67.1%) maintained SR, while 32.9% remained in AF. To better clarify the impact of SR restoration on FR, conducting a subgroup comparison between patients who maintained SR and those who experienced recurrent or persistent AF could more clearly elucidate the specific contribution of rhythm control to a reduction in FR.

Second, the authors classified FR severity into mild-to-severe categories based on the measured vena contracta width (VCW); however, this classification remains a qualitative assessment. We suggest employing quantitative parameters, such as regurgitant volume (RVol) or effective regurgitant orifice area (EROA), to more accurately quantify the degree of regurgitation. Additionally, although statistically significant reductions in left ventricular (LV), left atrial (LA), and right ventricular (RV) dimensions were observed post-TSA, these changes are close to the threshold of clinical measurement error inherent in transthoracic echocardiography. Having multiple examiners independently measure these parameters may minimize human measurement errors.

Third, relying on routine electrocardiograms at 12-month follow-up to assess rhythm status may lead to an overestimation of TSA efficacy, a limitation the authors themselves acknowledged. We propose adopting artificial intelligence-based wearable devices to improve AF detection accuracy. Additionally, we noted that post-TSA medical management strategies were not described for these patients. Whether medical therapies such as ACEI inhibitors, beta-blockers, or diuretics were administered during follow-up, particularly in cases of moderate-to-severe or severe FR, was not specified. Such medications could influence valvular regurgitation and potentially confound the assessment of the impact of rhythm control on FR.

In conclusion, we commend Sun *et al* for their innovative study evaluating thoracoscopic ablation for AF in patients with FR. Future research integrating biomarkers, advanced imaging techniques, and artificial intelligence might clarify the underlying mechanisms of AF and enable individualized ablation strategies, thereby improving patient prognosis.

## Data Availability

Data sharing is applicable to this article.

## Research registration unique identifying number (UIN)

Not required for this study.
